# Relation Between Rates of Geriatric Suicide and Consumption of Alcohol Beverages in European Countries

**DOI:** 10.1100/tsw.2006.71

**Published:** 2006-03-27

**Authors:** Leo Sher

**Affiliations:** Division of Neuroscience, Department of Psychiatry, Columbia University, New York, NY, USA

**Keywords:** human development, suicide, geriatric, alcohol, wine, beer, spirits, United States

## Abstract

Among older adults, suicide is a significant and persistent health problem. The highest suicide rate is found among white men aged 65 years and older. The causes of elder suicide are multifaceted. Although no predominate factor precipitates or explains geriatric suicide, alcohol is strongly linked to suicide attempts and completions. This study examined the relationship between rates of suicide in 65- to 74-year-olds and per capita consumption of alcoholic beverages in European countries. Data on suicide rates in 65- to 74-year-olds and per capita consumption of alcoholic beverages were obtained from the World Health Organization databases. Correlations were computed to examine relationships between suicide rates in 65- to 74-year-old males and females and per capita consumption of beer, wine, and spirits in the general population in 34 European countries. There was a positive correlation between suicide rates in 65- to 74-year-old males and per capita consumption of spirits. No correlations between suicide rates in 65- to 74-year-old males and per capita consumption of beer or wine were found. We also found no correlations between rates of suicide in 65- to 74-year-old females and per capita consumption of beer, wine, or spirits. The results of this study are consistent with reports that consumption of spirits is associated with suicide events. It is to be hoped that this paper will stimulate further studies that are necessary to clarify the relation between suicide rates in different age groups and consumption of alcoholic beverages, and attract more attention to the problem of geriatric suicide.

## INTRODUCTION

Suicide has constituted a critical public health problem for many decades. Among older adults, suicide is a significant and persistent health problem[1,2]. Indeed, the highest suicide rate is found among white men aged 65 years and older[3,4]. The causes of elder suicide are multifaceted[1,2,3,4,5]. Although no predominate factor precipitates or explains geriatric suicide, alcohol is strongly linked to suicide attempts and completions. Studies have evaluated the portion of older suicide decedents who had consumed alcohol immediately before the suicide event. A study of medical examiner toxicology reports on older suicide decedents found that 14.8% of 64- to 73-year-olds and 5.8% of those aged 74 and older had alcohol present in the blood stream at autopsy[6]. In a study of elder suicide attempts, 24.5% of patients aged 60–69 and 13% of those patients aged 70–79 had consumed alcohol before the attempt[7]. A number of reports suggest that alcohol sales and per capita consumption of alcoholic beverages may be related to suicidality[8,9,10]. This study examined the relationship between rates of suicide in 65- to 74-year-old males and females and per capita consumption of alcohol beverages in European countries.

## METHODS

Information on suicide rates in 65- to 74-year-old males and females and per capita consumption of beer, wine, and spirits in the general population in European countries was obtained from the World Health Organization (WHO) databases[11,12]. All European countries with a population more than 1 million people for which the WHO data were available were included in the study. The most recent available data were used. Correlations were computed to examine relationships between suicide rates in 65- to 74-year-old males and females and per capita consumption of beer, wine, and spirits in the general population in 34 European countries.

## RESULTS

Suicide rates in 65- to 74-year-olds and per capita consumption of alcoholic beverages in the general population in European countries are presented in Table 1. Suicide rates in 65- to 74-year-old males ranged from 3.7/100,000 in Albania to 94.5/100,000 in Russia. In 65- to 74-year-old females, suicide rates ranged from 2/100,000 in Greece to 25.1/100,000 in Slovenia. The mean suicide rate was 41.1 ± 26.2/100,000 in 65- to 74-year-old males, and 11.2 ± 5.7/100,000 in females. The mean per capita consumption of beer, wine, and spirits was 3.6 ± 2.2, 3.1 ± 2.2, and 3.4 ± 2.8 liters, respectively.

There was a positive correlation between suicide rates in 65- to 74-year-old males and per capita consumption of spirits ($n = 34$, $r = 0.4$, $p = 0.03$). No correlations between suicide rates in 65- to 74-year-old males and per capita consumption of beer or wine were observed ($n = 34$, $r = –0.1$, $p = 0.6$ and $n = 34$, $r = –0.1$, $p = 0.5$, respectively). We also found no correlations between rates of suicide in 65- to 74-year-old females and per capita consumption of beer, wine or spirits ($n = 34$, $r = –0.2$, $p = 0.4$; $n = 34$, $r = 0.03$; $p = 0.9$; and $n = 34$, $r = 0.2$, $p = 0.3$, respectively).

## DISCUSSION

The present study found that higher suicide rates in 65- to 74-year-old males were associated with higher per capita consumption of spirits in the general population of European countries. This finding is consistent with the results of the study that investigated the relationships between suicide rates and beverage-specific measures in the U.S. over periods of from 14–20 years[13]. It was found that suicide rates increased significantly as a function of increased spirit sales. Beer and wine sales were not associated with suicide rates. Probably, consumption of spirits is associated with suicide events. This can also be confirmed by the observation that after the legalization of strong beer in 1989 in Iceland, the decrease in consumption of spirits was accompanied by a decrease in the suicide rate[14].

At-risk drinking can be very common in the elderly. Paradoxically, when specific markers for alcohol problems are absent, psychophysical well-being is the dominant hallmark of at-risk drinking[15]. Therefore, when physicians' inquiry on alcohol use is restricted to intake information and does not assess the consequences of use, it cannot distinguish between safe and hazardous drinking.

Aging is associated with cognitive impairments[16]. Etiological models for alcohol use disorders have traditionally proposed trait and cognitive explanations for initiation, maintenance, and dependence[17]. Therefore, cognitive decline associated with aging may contribute to alcohol misuse in the older people.

**TABLE 1**
**Suicide Rates in 65- to 74-Year-Olds and Per Capita Consumption of Alcoholic Beverages in the General Population in European Countries**

| Country | Suicide Rates in 65- to 74-Year-Olds (per 100,000) | | Per Capita Alcohol Consumption (in Liters) | | |
|---|---|---|---|---|---|
| | Males | Females | Beer | Wine | Spirits |
| ALBANIA | 3.7 | 3.7 | 1 | 0.42 | 0.57 |
| AUSTRIA | 53.4 | 14.3 | 5.96 | 4.47 | 1.7 |
| BELARUS | 91 | 18.3 | 1.17 | 0.35 | 6.5 |
| BELGIUM | 35.5 | 13.6 | 5.4 | 2.88 | 1.36 |
| BOSNIA AND HERZEGOVINA | 12.9 | 6.5 | 1.25 | 0.2 | 4.89 |
| BULGARIA | 31.9 | 14.8 | 1.85 | 3.16 | 2.94 |
| CROATIA | 61.9 | 18.9 | 4.96 | 5.69 | 1.5 |
| CZECH REPUBLIC | 31.4 | 7.3 | 8.6 | 2.35 | 3.96 |
| DENMARK | 34.4 | 11.3 | 5.58 | 4.36 | 1.36 |
| ESTONIA | 62.9 | 11 | 3.86 | 1.01 | 3.15 |
| FINLAND | 35.8 | 10.6 | 4.41 | 2.57 | 2.73 |
| FRANCE | 38.8 | 13.6 | 2.1 | 8.25 | 2.96 |
| GERMANY | 29 | 10.8 | 6.82 | 3.27 | 2.38 |
| GREECE | 8.7 | 2 | 2.1 | 4.82 | 2.24 |
| HUNGARY | 78.5 | 20.1 | 3.55 | 4.37 | 3.64 |
| IRELAND | 19.1 | 0.8 | 8.8 | 4.35 | 2.65 |
| ITALY | 17.9 | 5.7 | 1.47 | 7.11 | 0.58 |
| LATVIA | 75.7 | 16.1 | 1.94 | 0.7 | 6.71 |
| LITHUANIA | 89.8 | 19.7 | 5.53 | 1.84 | 4.92 |
| MACEDONIA | 21.8 | 14.4 | 0.64 | 1.28 | 2.9 |
| MOLDOVA | 40.7 | 10 | 0.35 | 3.09 | 15.17 |
| NETHERLANDS | 15 | 9 | 4.62 | 2.72 | 2.05 |
| NORWAY | 19.9 | 8.1 | 2.84 | 1.33 | 0.83 |
| POLAND | 34.3 | 7.8 | 2.99 | 0.9 | 4.38 |
| PORTUGAL | 38.8 | 8 | 3.87 | 7.22 | 5.27 |
| ROMANIA | 33 | 7.7 | 3.22 | 3.39 | 1.31 |
| RUSSIA | 94.5 | 18.2 | 1.67 | 1.07 | 8.05 |
| SLOVAKIA | 28.4 | 4.1 | 4.95 | 1.75 | 5.37 |
| SLOVENIA | 88.7 | 25.1 | 3.76 | 4.66 | 3.3 |
| SPAIN | 21.3 | 7.1 | 3.76 | 4.61 | 2.79 |
| SWEDEN | 29.5 | 9.4 | 3.29 | 2.19 | 1.23 |
| SWITZERLAND | 38.7 | 15.1 | 3.25 | 6.43 | 1.77 |
| UKRAINE | 72.1 | 13.5 | 0.74 | 0.25 | 2.77 |
| UNITED KINGDOM | 8.7 | 3.4 | 5.46 | 2.29 | 1.89 |

*Conversion factors used to estimate amount of pure alcohol in (barley) beer is 5.0%, wine 12%, and spirits 40% of alcohol (other conversion factors were used for some types of beer and other beverages).

The results of this epidemiological study should be treated with caution because of some limitations: (1) per capita consumption of spirits in the general population may be different from per capita consumption of spirits in 65- to 74-year-olds and (2) this study does not take into account cultural differences and differences in drinking patterns between countries.

The present study found a relation between suicide rates in 65- to 74-year-old males and consumption of spirits in the general population. It is to be hoped that this paper will stimulate further studies that are necessary to clarify the relation between suicide rates in different age groups and consumption of alcoholic beverages, and attract more attention to the problem of geriatric suicide.

## RERERENCES

1. Carney, S.S., Rich, C.L., Burke, P.A., and Fowler, R.C. (1994) Suicide over 60: the San Diego study. *J. Am. Geriatr. Soc*. **42**, 174–180.
2. Conwell, Y. (2001) Suicide in later life: a review and recommendations for prevention. *Suicide Life Threat. Behav. (Suppl)* **31**, 32–47.
3. Pearson, J.L. and Conwell, Y. (1995) Suicide in late-life: challenges and opportunities for research. *Int. Psychogeriatr*. **7**, 131–136.
4. Sher, L. (2004) Preventing suicide. *QJM* **97**, 677–680.
5. Pearson, J.L. and Brown, G.K. (2000) Suicide prevention in later life: directions for science and practice. *Clin. Psychol. Rev*. **20**, 685–705.
6. Conwell, Y., Rotenberg, M., and Caine, E.D. (1990) Completed suicide at age 50 and over. *J. Am. Geriatr. Soc*. **38**, 640–644.
7. Frierson, R.L. (1991) Suicide attempts by the old and the very old. *Arch. Intern. Med*. **151,** 141–145.
8. Sher, L. (2005) Alcohol use and suicide rates. *Med. Hypoth*. **65**, 1010–1012.
9. Norstrom, T. and Skog, O.J. (2001) Alcohol and mortality: methodological and analytical issues in aggregate analyses. *Addiction* **96(Suppl 1),** S5–17.
10. Smith, D.I. and Burvill, P.W. (1991) Relationship between alcohol consumption and attempted suicide morbidity rates in Perth, Western Australia, 1968–1984. *Addict. Behav*. **16(1–2),** 57–61.
11. World Health Organization. Mental Health. Country Reports and Charts. http://www.who.int/mental_health/prevention/suicide/country_reports/en/ Accessed: January 9, 2006.
12. World Health Organization. Adult per capita alcohol consumption. http://www3.who.int/whosis/alcohol/alcohol_apc_data.cfm?path=whosis,topics,alcohol,alcohol_apc,alcohol_apc_data&language=english Accessed: July 27, 2004 and January 9, 2006.
13. Gruenewald, P.J., Ponicki, W.R., and Mitchell, P.R. (1995) Suicide rates and alcohol consumption in the United States, 1970–89. *Addiction* **90,** 1063–1075.
14. Lester, D. (1999) Effect of changing alcohol laws in Iceland on suicide rates. *Psychol. Rep*. **84(3 Pt 2),** 1158.
15. Di Bari, M., Silvestrini, G., Chiarlone, M., De Alfieri, W., Patussi, V., Timpanelli, M., Pini, R., Masotti, G., and Marchionni, N. (2002) Features of excessive alcohol drinking in older adults distinctively captured by behavioral and biological screening instruments: an epidemiological study. *J. Clin. Epidemiol*. **55**, 41–47.
16. Comijs, H.C., Dik, M.G., Aartsen, M.J., Deeg, D.J., and Jonker, C. (2005) The impact of change in cognitive functioning and cognitive decline on disability, well-being, and the use of healthcare services in older persons. Results of Longitudinal Aging Study Amsterdam. *Dement. Geriatr. Cogn. Disord.* **19(5–6),** 316–323.
17. Anderson, K.G., Schweinsburg, A., Paulus, M.P., Brown, S.A., and Tapert, S. (2005) Examining personality and alcohol expectancies using functional magnetic resonance imaging (fMRI) with adolescents. *J. Stud. Alcohol* **66**, 323–331.

## BIOSKETCH

**Leo Sher, MD** is a psychiatrist with a background in internal medicine. His areas of research and clinical expertise include neurobiology; diagnosis and treatment of mood, anxiety, and personality disorders; alcoholism; and suicidal behavior. He graduated Summa Cum Laude from the Ukrainian National Medical University in Kiev, Ukraine; did his residency in psychiatry at the Albert Einstein College of Medicine Program at Long Island Jewish Medical Center in New York and the National Institute of Mental Health Program in Bethesda, Maryland. He completed a Research Fellowship at the National Institute of Mental Health in Bethesda, Maryland. Currently, Assistant Clinical Professor of Psychiatry at Columbia University and Research Psychiatrist in the Department of Neuroscience at the New York State Psychiatric Institute in New York City. He has authored more than 250 scientific publications, has been a reviewer for numerous medical journals, the recipient of several awards, and the first researcher in North America who introduced the use of the combined dexamethasone suppression/corticotropin-releasing hormone stimulation test for psychiatric purposes. E-mail: LS2003@columbia.edu Website: http://asp.cumc.columbia.edu/facdb/profile_list.asp?uni=ls2003&DepAffil=Psychiatry
